# Potential Cost-Effectiveness of RSV Vaccination of Infants and Pregnant Women in Turkey: An Illustration Based on Bursa Data

**DOI:** 10.1371/journal.pone.0163567

**Published:** 2016-09-30

**Authors:** Koen B. Pouwels, Sefika E. Bozdemir, Selen Yegenoglu, Solmaz Celebi, E. David McIntosh, Serhat Unal, Maarten J. Postma, Mustafa Hacimustafaoglu

**Affiliations:** 1 Unit of PharmacoEpidemiology & PharmacoEconomics, Department of Pharmacy, University of Groningen, Groningen, The Netherlands; 2 Modelling & Economics Unit, Centre for Infectious Disease Surveillance & Control, Public Health England, London, United Kingdom; 3 Division of Pediatric Infectious Diseases, Uludağ University Faculty of Medicine, Bursa, Turkey; 4 Department of Pharmacy Management, Hacettepe University Faculty of Pharmacy, Ankara, Turkey; 5 Department of Medicine, Imperial College London, London, United Kingdom; 6 Department of Infectious Diseases and Clinical Microbiology, Hacettepe University Faculty of Medicine, Ankara, Turkey; 7 Institute of Science in Healthy Aging & healthcaRE (SHARE), University Medical Center Groningen (UMCG), Groningen, The Netherlands; 8 Department of Epidemiology, UMCG, University of Groningen, Groningen, The Netherlands; University of Tennessee Health Science Center, UNITED STATES

## Abstract

**Background:**

Worldwide, respiratory syncytial virus (RSV) is considered to be the most important viral cause of respiratory morbidity and mortality among infants and young children. Although no active vaccine is available on the market yet, there are several active vaccine development programs in various stages. To assess whether one of these vaccines might be a future asset for national immunization programs, modeling the costs and benefits of various vaccination strategies is needed.

**Objectives:**

To evaluate the potential cost-effectiveness of RSV vaccination of infants and/or pregnant women in Turkey.

**Methods:**

A multi-cohort static Markov model with cycles of one month was used to compare the cost-effectiveness of vaccinated cohorts versus non-vaccinated cohorts. The 2014 Turkish birth cohort was divided by twelve to construct twelve monthly birth cohorts of equal size (111,459 new-borns). Model input was based on clinical data from a multicenter prospective study from Bursa, Turkey, combined with figures from the (inter)national literature and publicly available data from the Turkish Statistical Institute (TÜÏK). Incremental cost-effectiveness ratios (ICERs) were expressed in Turkish Lira (TL) per quality-adjusted life year (QALY) gained.

**Results:**

Vaccinating infants at 2 and 4 months of age would prevent 145,802 GP visits, 8,201 hospitalizations and 48 deaths during the first year of life, corresponding to a total gain of 1650 QALYs. The discounted ICER was estimated at 51,969 TL (26,220 US $ in 2013) per QALY gained. Vaccinating both pregnant women and infants would prevent more cases, but was less attractive from a pure economic point of view with a discounted ICER of 61,653 TL (31,106 US $ in 2013) per QALY. Vaccinating only during pregnancy would result in fewer cases prevented than infant vaccination and a less favorable ICER.

**Conclusion:**

RSV vaccination of infants and/or pregnant women has the potential to be cost-effective in Turkey. Although using relatively conservative assumptions, all evaluated strategies remained slightly below the threshold of 3 times the GDP per capita.

## Introduction

In various regions of the world, respiratory syncytial virus (RSV) is a common causative agent of childhood acute bronchiolitis and pneumonia, with frequencies varying from 12 to 52% [1]. In hospitalized infants and children <2 years of age, RSV causes up to 50–90% of acute bronchiolitis and 5–40% of pneumonia cases [2], [3]. Premature infants and children <2 years of age with underlying chronic lung disease or congenital heart disease have a high risk of hospitalization and sequelae [4]. A study from the United Kingdom, estimated that 14% and 3% of hospitalizations in those <2 years of age are due to RSV and influenza virus, respectively [5]. There is also evidence that bronchiolitis cases are more severe when RSV is a causative agent, thereby further increasing the burden. In a multicenter study conducted in Finland, of the hospitalized infants with bronchiolitis <2 years of age, the length of stay for RSV bronchiolitis was longer than for other causes and ICU admissions (10%) were higher than for other causes [6]. Globally, RSV causes nearly 34 million lower respiratory tract infection (LRTI)–acute bronchiolitis or pneumonia—episodes and 3.4 million hospitalizations per year in infants and children <5 years of age [7].

In Turkey, RSV is also a common cause of LRTI among infants and children. In a prospective study, 8% of all babies <6 months developed bronchiolitis caused by RSV and half of them (4%) were hospitalized [8]. In studies from different regions of Turkey among children hospitalized with LRTI, RSV was identified as a causative agent in 35–50% of the cases [8], [9], [10]. In the Bursa region, the annual incidence of hospitalization due to RSV+ LRTI was found to be 7.81 per 1000 infants and children <2 years of age. Compared to other age groups, the burden was highest in children aged 0–3 months, 48% of RSV+ LRTI hospitalizations were in this age-group [9].

Vaccination has the potential to be the most effective approach for reducing the global burden of disease associated with RSV infections, and the development of a safe and effective vaccine may have an important role in decreasing the burden of RSV infections especially in infants. However, there is no commercially available active vaccine for RSV infections. Currently, there are several active vaccine development programs at various stages [11].

Given the high burden among infants and young children, an intuitive strategy would be vaccination of infants. Vaccination of pregnant women is another potential strategy to protect full-term infants from RSV disease during the first few months following birth, by means of transplacental transfer of antibodies [12]. A study from Turkey found that the risk of RSV LRTIs was high among babies having low maternal antibody levels (<20RU) [8], suggesting that maternal vaccination may be an option to decrease the substantial burden among infants and the very young children.

To analyze whether any one of the RSV-vaccines in development might be an asset for national immunization programs, modeling the costs and benefits of various vaccination strategies is needed [12]. This cost-effectiveness study aims to evaluate the economic burden of RSV infection among infants and children <2 years in Turkey, and to assess the potential public health and economic benefits of RSV vaccination. Different vaccination strategies are evaluated, thereby assessing the cost-effectiveness of vaccination of pregnant women and/or young infants and exploring the potential impact of seasonal vaccination.

## Material and Methods

### Study population

This cost-effectiveness analysis is based on a multicenter prospective study from Bursa (Turkey), combined with figures from the (inter)national literature and publicly available data from the Turkish Statistical Institute [13].

Details about the Bursa study have been described elsewhere [9]. In brief, the study was a multicenter prospective study, in which all infants and children <2 years of age who were hospitalized for LRTI in the three largest hospitals of Bursa City Center (Uludağ University, Pediatric Infectious Diseases Department, Dörtçelik Children’s Hospital, and Doruk Private Bursa Hospital) between 1 March 2010 and 28 February 2011 (12 months) were tested for RSV positivity. Patients who had received in the previous 10 days or were receiving palivizumab prophylaxis or intravenous immunoglobulin at the time of admission were excluded from the study. RSV status was determined by an experienced physician or a trained nurse in the first 48 hours of hospitalization with RSV Respi-Strip (Coris Bioconcept Organization). Hospitalization costs were obtained from the documented hospital bills after discharge.

The data from this Bursa study were used to parameterize the model regarding the incidence of RSV-associated hospitalizations and related costs, thereby assuming that these figures are representative for the whole of Turkey. Since patients receiving palivizumab or intravenous immunoglobulin were excluded from that study, and accurate data about the number of children with high-risk conditions, e.g. congenital heart disease and/or chronic heart disease, in Turkey are lacking, we did not model an impact of vaccination on palivizumab or immunoglobulin use.

### Model design and methodological assumptions

We constructed a static multi-cohort Markov model with cycles of one month using Microsoft Office Excel 2010 to compare the cost-effectiveness of vaccinated cohorts versus non-vaccinated cohorts. The 2014 Turkish birth cohort was divided by twelve to construct twelve monthly birth cohorts of equal size (111,459 new-borns) (Turkish Statistical Institute) [13].

The cohorts were followed in monthly cycles until their 2nd birthday, and clinical and economic effects were subsequently compared. Hence the time-horizon was 2 years. In scenarios where RSV-associated mortality was modelled and vaccination was assumed to reduce this burden, the cycles still stopped at the 2nd birthday of the birth cohorts. However, for these scenarios, discounted life-time years lost were included in the calculation of the incremental cost-effectiveness ratio.

In our primary analysis we compared the cost-effectiveness of three different vaccination strategies to the current situation of no vaccination: 1) vaccinating infants at 2 and 4 months of age, 2) vaccinating pregnant women and 3) vaccinating pregnant women and infants at 2 and 4 months of age. We assumed that vaccinating pregnant women would occur during the third trimester and that protection of the child started at birth, with no additional protection to the pregnant women given the lack of reliable data about the burden RSV among pregnant women. These are both conservative assumptions: recent animal studies suggest that RSV may be vertically transmitted, after which the virus may interfere with crucial developmental processes [14]; although poorly documented, maternal RSV infection may lead to clinically severe maternal disease [15].

In our model, all individuals of the unvaccinated cohort start as susceptible to symptomatic RSV infection requiring a general practitioner (GP) visit. Each month the cohort faces a risk of a symptomatic RSV infection requiring a GP visit. The risk is dependent on both the age of the cohort (in months) and the season (in calendar months). A proportion of those GP-visits will also lead to a hospitalization, of which a proportion will die due to the RSV infection. Although re-infection with RSV occurs frequently, re-infections usually have a mild character with symptoms of uncomplicated upper respiratory tract infection [16]. Hence, we assumed that after RSV-related GP visits patients moved to an immune state up until their 2nd birthday. Dependent on the vaccination scenario, individuals of the vaccinated cohort face a monthly probability of being vaccinated. Dependent on the assumed vaccine effectiveness, a proportion of the vaccinated subjects move to the immune state. Some of the subjects will become susceptible again due to waning of the vaccine-induced immunity. Finally, individuals can die in all states of the model due to other causes than RSV. Schematic representation of the Markov model and the corresponding RSV-related GP visits, hospitalizations and death can be found in the supplementary files (S1 Appendix).

### Health-care utilization

The point estimates and probability distributions used to parameterize the model are shown in Table 1. Data from the Bursa study were used to estimate the transition probability for hospitalizations [9]. The anonymized electronic data of this study were used to calculate age- and calendar-month specific transition probabilities. To remove random fluctuation due to a limited amount of hospitalizations at higher ages, we used the actual numbers for the months 0–3, a 3-month moving average from 4–11 months, and for the months 12–23 we used the average amount of cases observed per month during the second year of life. We considered using age-distribution observed in larger studies from different countries [17], [18], [19], however those studies appeared to have a relevantly different pattern in the incidence during the crucial first 4 months of life. The incidence was adjusted for imperfect sensitivity (91%) and specificity (98%) of the Respi-Strip that was used in the Bursa study [20], using the following formula:

$$\frac{observed\;incidence-\left(1-specificity\right)}{senstivity+specificity-1}$$

**Table 1:** Disease and vaccine parameters used in the economic model.

	Base-case	Distribution	References
Epidemiological parameters			
RSV-hospitalizations	^#^	^#^	
RSV-related GP visits (proportion of hosp)	17.78	Beta (94.32; 1,582.46)	[21]
ICU admission (as proportion of hosp)	0.10	Beta (26;228)	[9]
RSV-related mortality 0–11 m (proportion of hosp)	0.0068	Beta (99.32;1,4536.27)	[9], [22], [25]
RSV-related mortality 12+ m (proportion of hosp)	0.00026	Beta (99.97;390,299.03)	[9], [22], [25]
Quality of life parameters			
Disutility GP-treated RSV infection	0.16	Beta (56.30;281.40)	[26], [27]
Disutility hospital-treated RSV infection	0.43	Beta (117.18;154.38)	[26], [27]
Duration of disutility RSV infections (days)	14	Fixed	[26], [27]
Average quality of life (varying with age in years)			[32], [34]
<16 y	0.88	Beta (8.41;1.15)	
16–24 y	0.80	Beta (11.97;2.98)	
25–34 y	0.80	Beta (11.97;2.98)	
35–44 y	0.77	Beta (12.75;3.72)	
45–54 y	0.75	Beta (13.35;4.50)	
55–64 y	0.70	Beta (14.04;6.15)	
65–74 y	0.69	Beta (14.09;6.44)	
75+ y	0.64	Beta (14.11–7.86)	
Vaccine parameters			
Vaccination coverage	0.85	Fixed	Assumed
Effectiveness vaccination pregnant women	0.6	Fixed	Assumed
Effectiveness vaccination infants 2 months of age	0.6	Fixed	Assumed
Effectiveness vaccination infants 4 months of age	0.75	Fixed	Assumed

GP: general practitioner, hosp: hospitalization, ICU: intensive care unit, m: month, RSV: respiratory syncytial virus, y: years.

^#^ Age- and calendar-month dependent.

There was no data on the actual number of RSV infections not resulting in health-care utilization and RSV infections resulting in a GP visit.. To obtain the number of RSV-related GP visits, we assumed that the proportion of RSV-related GP visits resulting in RSV-related hospitalizations would be the same as in the Netherlands [21]. Therefore, we estimated that for 17.78 RSV-related GP visits there will be one RSV-related hospitalization.

### Mortality

Published estimates of RSV-related mortality among children <12 months old vary substantially from 0 up to 22 per 100,000 person-years [21], [22], [23], [24], [25]. We used the US mortality rate as estimated by Thompson *et al*., in the absence of Turkey-specific data. That study estimated the RSV-related mortality rate at 5.3 per 100,000 persons for children aged <12 months, which in our model results in a case fatality ratio for children admitted to the hospital of 5.3/781 = 0.7%. This is very similar to the average case fatality ratio of 0.7% for children aged <12 months admitted to the hospital with RSV-associated LRTI in industrialized countries [25].

### Utilities (QALYs)

Quality of life losses were included during the acute disease episode. Although several cost-utility analyses included quality of life losses during the acute phase of RSV disease, no study so far incorporated actual utility losses estimated specific for the acute phase of RSV disease. After systematically reviewing the literature, we found only one study estimating utility weights specific for this phase of the disease [26], [27]. That study estimated disutility for 4 different states using the time-tradeoff (TTO) methodology: 1) mild RSV disease resulting in emergency room or family doctor’s office; 2) moderate RSV disease resulting in hospitalization; 3) severe RSV disease resulting in intensive care unit admission with oxygen support by mask-assisted breathing; and 4) very severe RSV disease resulting in intensive care unit admission with oxygen support by breathing machine. Assuming the same average duration of infection of 14 days, we based the QALY loss due to an RSV-associated GP visits on the estimate for ‘mild RSV disease’. The QALY loss due to an RSV-associated hospitalization was based on the estimates for moderate RSV and very severe RSV disease, thereby using the same risk of ICU-admission as observed in the Bursa study (10.2%). It should be noted that these estimates are obtained by a proxy questionnaire, i.e. adults were presented with the 4 different hypothetical scenarios in children [27].

In scenarios where we assumed that vaccination would prevent potential RSV-associated mortality, we also included quality of life losses due to death. Because RSV-associated mortality may be mainly found in high-risk patients with a likely reduced baseline quality of life and life-expectancy [28], we assumed that all RSV-associated deaths would occur among patients with a reduced quality of life and life-expectancy. Similar to studies evaluating the cost-effectiveness of palivizumab among high-risk infants [29], [30], we calculated the life expectancy for high-risk children based on a study evaluating the survival of infants with CHD in the United Kingdom [31]. Based on that study we assumed that 95.3% of children with CHD would survive to age 16 years if they had survived to age of one year. We assumed that the survival after that age would be the same as for the general population [13], resulting in a life-expectancy of 75.0 years at the age of one year. For comparison, the life expectancy for the general population at this age is 77.9 years.

The assumed reduced baseline quality of life of infants that die due to RSV was based on a study comparing quality-of-life in children with a history of preterm birth and RSV hospitalization compared with a control group of preterm children without a history of RSV hospitalization [32]. That study assessed the children’s health related quality of life at the age of 5, using the Health Utilities Index (HUI) Mark 2. The median HUI 2 multi-attribute utility function was 0.88 in the children with a proven RSV infection, while the median was 0.95 in the control group. In line with our assumption that the association between RSV and asthma is not causal, we assumed that RSV does not confer long-term sequelae. Therefore we conservatively multiplied all baseline quality of life estimates of the general population with 0.88 to obtain the assumed reduced quality of life in children dying due to a RSV infection. In the absence of Turkish data, similar to another recent Turkish cost-effectiveness analysis [33], age-specific population norm quality of life estimates were based on UK data [34].

### Costs

Cost estimates (Table 2) were derived from documented hospital bills after discharge in the Bursa study and published sources. Analyses were conducted from the societal perspective, including productivity costs. All costs were reported in 2013 Turkish Lira (TL) (1 TL = 0.50 US $ in 2013). Costs not available at 2013 price levels were inflated using the Turkish consumer price index. We used the same cost for GP visits and productivity losses as in a recent study evaluating the cost-effectiveness of rotavirus vaccination in Turkey [35]. The vaccine price was assumed to be 60 TL.

**Table 2:** Cost parameters used in the economic model.

	Base-case	Distribution	References
Cost of GP visit (TL)	38	Fixed	[35]
Cost of RSV-hospitalization (TL)	1,992.04	Gamma (1;1,992.04)	[9]
Proportion of woman working	0.15	Triangular (0.1;0.15;0.2)	[35]
Productivity loss per GP visit (workdays)	1	Triangular (0;1;2)	[35]
Productivity loss per hospitalization (workdays)	3	Triangular (1;3;5)	[35]
Cost of lost workday (TL)	28.49	Gamma (1;28.49)	[35]
Price of vaccine per dose (TL)	60	Fixed	Assumed

GP: general practitioner, RSV: respiratory syncytial virus, TL: Turkish Lira.

### Outcome measures and cost-utility analysis

The main outcome measure was the incremental cost-effectiveness ratio (ICER) expressed as costs per quality-adjusted life year (QALY) gained (also sometimes referred to as the incremental cost-utility ratio). The ICER was calculated by dividing the difference in cost between the two alternatives by the difference in their effect. It represents the average incremental cost in TL associated with 1 QALY gained. ICERs were calculated for the different vaccination schedules compared to no vaccination. Besides ICERs, the model also tracked the number of GP visits, hospitalizations, and deaths due to RSV, together with associated costs and QALY losses. Cost and health outcomes were discounted at 3% per year.

### Vaccine effectiveness

Because RSV-vaccines are still under development, assumptions had to be made regarding the vaccine effectiveness. As explained above, we considered vaccination at three different moments: during the third trimester of pregnancy, and at 2 and 4 months of age in infants. In our base case scenarios we assumed vaccination coverage of 85% for all vaccinations. The initial vaccine efficacy of vaccination during pregnancy was assumed to be 60%. The first infant dose at 2 months of age was assumed to keep the vaccine effectiveness at 60%. The second infant dose at 4 months of age was assumed to raise the vaccine effectiveness to 75% and provide this level of protection until the date the vaccinated child became 2 years old.

Potential herd protection effects were not included, because of insufficient data to inform and calibrate such a model for Turkey. Moreover, transmission dynamic modelling studies from Kenya indicate that vaccination against RSV during pregnancy or in infants does not likely infer a substantial herd protection effect [36], [37].

### Sensitivity analyses

Univariate, multivariate, scenario, and probabilistic sensitivity analyses were performed to explore parameter uncertainty. Probabilistic sensitivity analyses were performed to evaluate the uncertainty of the ICERs taking into account uncertainty across all parameters simultaneously.

Transition probabilities and disutilities were inserted as beta distributions. Cost-related parameters were inserted as fixed values when prices were fixed (vaccination costs, GP visits) and as gamma distribution when they were estimated from a sample (hospitalizations). For parameters for which the uncertainty had to be obtained by expert opinion, triangular distributions were used. Outcome values were generated by running the model 5,000 times. The decision uncertainty, i.e. the probability that the vaccination strategy of interest is cost-effective for different threshold of the willingness to pay per QALY gained, is subsequently presented by cost-effectiveness acceptability curves. In addition, these simulations were used to generate 95% uncertainty intervals around the ICERs.

In the univariate analysis, all relevant parameters were varied with 25% to explore the impact of each parameter relative to each other, while holding all other parameters fixed.

We also estimated the potential cost-effectiveness of seasonal vaccination as this may be associated with a substantial decrease in the total vaccination costs. Given the seasonality of RSV observed in Bursa, we considered vaccination of all children aged between 2 and 6 months in December until February, thereby assuming a vaccine effectiveness of 60% and duration of protection of 5 months.

### Ethics Statement

Ethics committee approval was received for this study from the ethics committee of Uludağ University Medical Faculty (number: 2010-2/34). The observational study the RSV hospitalization rates were based on was not registered in a public database. Anonymized electronic data of that study were used to calculate age- and calender-month specific transition probabilities.

## Results

Without vaccination, RSV infection among children aged 0–2 years old were modelled to cause 343,711 GP visits, 19,334 hospitalizations and 118 deaths in a Turkish birth-cohort followed for 2 years. The total number of discounted QALYs lost would be 5,243, of which 56% was due to deaths and 44% due to complications with related hospitalizations and GP visits. In this timeframe, the total discounted cost mounted up to 53,123,447 TL, of which 3% was due to productivity losses by parents.

Table 3 summarizes the impact of the three different vaccination strategies (infants at 2 and 4 months of age; pregnant women + infants at 2 and 4 months of age; pregnant women only), using base case assumptions.

**Table 3:** Impact and incremental cost-effectiveness ratios of vaccination strategies.

Vaccination	GP visits	Hospitalizations	Deaths	ICER (TL/QALY)
None	343,711	19,334	118	-
2+4 m infant	197,909	11,132	70	51,969 (95% CI 35,313–68,244)
Pregnancy	285,693	16,070	95	60,638 (95% CI 45,154–76,806)
Pregnancy + 2+4m infant	157,348	8,851	54	61,653 (95% CI 44,347–79,799)

GP: general practitioner, ICER: incremental cost-effectiveness ratio, m:month, QALY: quality adjusted life-year, TL: Turkish Lira.

Vaccinating infants at 2 and 4 months of age would prevent 145,802 (95% CI 123,780–169,393) GP visits, 8,201 (95% CI 7,849–8,526) hospitalizations and 48 (95% CI 38–57) deaths during the first year of life, corresponding to a total gain of 2,172 (95% CI 1,836–2,508) discounted QALYs. Incremental costs of this 2-dose infant schedule would be approximately 112 million (95% CI 81–144 million) TL. The discounted ICER was estimated at 51,969 (95% CI 35,313–69,244) TL per QALY gained, which is below the threshold of 3x GDP per capita in Turkey (61,821 TL) [13]. Fixing all other parameters and assuming the same effectiveness for both infant doses, the vaccine effectiveness should be at least 61% to remain below this threshold. If the vaccine price would be reduced or increased by 25%, the vaccine effectiveness should be at least 46% or 75%, respectively.

Vaccinating both pregnant women and infants at 2 and 4 months of age would prevent an additional 40,560 (95% CI 33,976–47,849) GP visits, 2,282 (95% CI 2,217–2,339) hospitalizations and 16 (95% CI 13–19) deaths. However, this vaccination strategy was, although still below the threshold of 61,821 TL, less attractive from a pure economic point of view with a discounted ICER of 61,653 (95% CI 44,347–79,799) TL per QALY. If the vaccine price would be reduced or increased by 25%, and assuming an equal effectiveness for all vaccinations, the vaccine effectiveness should be at least 52% or 83%, respectively.

Vaccinating only during the third trimester of pregnancy resulted in an ICER of 60,638 (95% CI 45,154–76,806) TL per QALY. Although, vaccinating only during pregnancy was associated with much less total vaccination costs than an infant schedule with vaccinations at 2 and 4 months of age (68 vs 135 million TL), the cost-effectiveness of the infant vaccination was more favorable because this schedule would reduce the burden of RSV more substantially. Obviously, when the duration of protection would be longer after vaccination during pregnancy, more favorable ICERs could be obtained with a pregnancy only schedule. For example, if vaccination during pregnancy would protect infants for 5 instead of 3 months the ICER would be 35,425 (95% CI 21,342–49,915) TL per QALY, while preventing 91,126 (95% CI 78,024–105,305) GP visits, 5,126 (95% CI 4,869–5,359) hospitalizations and 36 (95% CI 29–43) deaths compared to no vaccination.

### Sensitivity analysis

**Fig 1:**
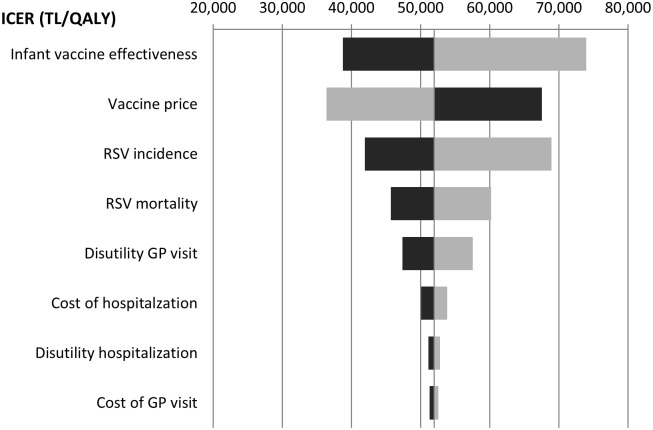
Univariate sensitivity analysis for infant vaccination at 2 and 4 months of age. The parameters are varied 25% in both ways (dark bars: 25% increase, light bars: 25% decrease). GP: general practitioner, ICER: incremental cost-effectiveness ratio, RSV: respiratory syncytial virus.

Univariate sensitivity analyses showed that when varying parameters estimates by 25% in both ways, assumed vaccine effectiveness, RSV incidence, and the assumed vaccine price were the major determinants of the ICER (Fig 1). Other important variables were the RSV-related mortality, and the disutility associated with a GP visit. The disutility associated with a hospitalization was much less important, which can be explained by the fact that hospitalizations were much less common than GP visits in our model.

Univariate sensitivity analysis for the other vaccination schedules showed similar sensitivity to parameters, except that the vaccine effectiveness of pregnancy vaccination also played a role in these scenarios. For those latter scenarios the duration of protection after vaccination during pregnancy is a major determinant of the cost-effectiveness.

Seasonal vaccination of all children aged between 2 and 6 months between December and February resulted in an ICER of 23,965 TL per QALY, while preventing 71,729 GP visits, 4,009 hospitalizations and 28 deaths.

### Probabilistic sensitivity analysis

**Fig 2:**
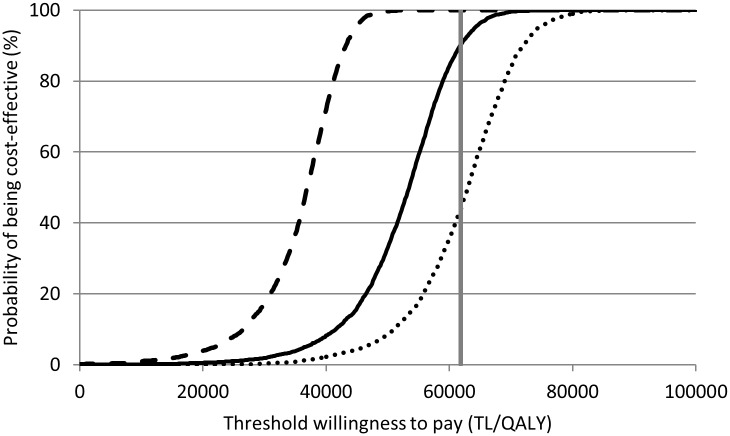
Cost-effectiveness acceptability curves. Cost-effectiveness acceptiblity curves for 2+4 months infant vaccination (solid line), vaccination of pregnant women (dotted line) and vaccination of pregnant women + infant vaccination (dashed line). The grey vertical line indicates the 3x GDP per capita threshold (61,821 TL per QALY gained).

Fig 2 shows the cost-effectiveness acceptability curves obtained via probabilistic sensitivity analyses. The curves show the likelihood that the vaccination strategy of interest is cost-effective at different thresholds of the willingness to pay per QALY gained.. At a threshold of 61,821 TL, 90%, 51% and 44% of Monte Carlo samples are considered cost-effective for infant vaccination only, pregnant women vaccination only and combination of both strategies, respectively. This indicates that, when taking into account uncertainty in parameter estimates, vaccination of infants with a vaccine that has similar properties as assumed in our base-case has a high probability of being cost-effective.

## Discussion

Our analysis shows that protection of infants and young children against RSV using an effective vaccine has the potential to be cost-effective in Turkey. Taking into account both the potential impact on the epidemiology and the potential cost-effectiveness of different vaccination strategies, a 2-dose infant vaccination schedule alone or in combination with vaccination of pregnant women during their third trimester seems to have most potential.

Vaccinating of pregnant women only becomes much more attractive from both an economical and epidemiological point of view if the duration of protection is sufficiently long as shown in our sensitivity analysis. In addition, inclusion of direct protection among pregnant women in the model may substantially increase the cost-effectiveness. Influenza vaccination of pregnant women has previously been estimated to be potentially cost-effective even without protection of infants [38]. However, there is a lack of reliable data about the burden of RSV among pregnant women. Nevertheless, some recent animal studies suggest that RSV may be vertically transmitted, after which the virus may interfere with crucial developmental processes of the fetus [14] and although poorly documented, maternal RSV infection may lead to clinically severe maternal disease [15].

This is the first study that evaluated the potential cost-effectiveness of RSV vaccination in a middle-income country. We considered various vaccination strategies, including vaccination of pregnant women and/or infants and seasonal vaccination.

Our study has several potential limitations. First, similar to previous studies [21], [39], we decided to use a static model in the absence of reliable data to inform and calibrate such a model. Hence, we did not include potential herd immunity or potential age-shifts. Recently, two transmission dynamic models assessed the potential epidemiological impact of RSV vaccination in Kenya [36], [37]. The availability of data on transmission patterns in Kenya allowed those researchers to better inform and calibrate the models to actual data. Nevertheless, both studies came to different conclusions: Poletti *et al*. found that annual vaccination of all primary school children is the only alternative strategy to immunization of infants [36], while Kinyanjui *et al*. concluded that immunization of young children aged 5-10m was an effective method of protection of infants against hospitalizations [37]. These contrasting results and the substantial uncertainty around the estimates of both studies illustrate that more data is needed before a reliable transmission dynamic model can be used for a health economic evaluation of different vaccination strategies [40]. However, if it can be proven that the vaccine has an effect on transmission of the virus, a transmission dynamic model is needed to adequately capture all benefits from different vaccination strategies, including vaccination of the elderly [41], [42].

Second, similar to previous studies [21], [39], we only included primary RSV infections, which may have led to an underestimation of the cost-effectiveness. However, reinfections tend to be relatively mild.

Third, we conservatively did not include RSV-associated asthma in our model. A recent meta-analysis suggests that RSV-infection increases the risk of asthma [43]. However, it is also possible that RSV hospitalizations do not cause subsequent asthma, but merely identify those patients who are predisposed to develop asthma, regardless of the severity of the RSV infection. Moreover, studies that do find an increased risk tend to show that the association decreases with age of the children [43]. This suggests that either the asthma-symptoms caused by RSV hospitalization are temporarily; or that RSV hospitalizations do increase the risk of asthma only in children which such a high propensity of developing asthma that they will inevitably develop asthma later in life due to some other environmental factor; or that the RSV hospitalizations just lead to an earlier diagnosis of asthma without being a causal risk factor. A recent randomized controlled trial found that wheezing during the first year of life could be reduced among healthy infants by providing palivizumab treatment [44]. However, more research is needed to prove a causal relationship between RSV and asthma.

Fourth, in the absence of reliable data we conservatively did not model a reduction in antibiotic usage for respiratory infections [41], [45], of which a large proportion is caused by RSV in this age-group [1], [41], [46]. Given the current concerns about rises in antibiotic resistance rates worldwide [47], this could be an important factor to take into account in future analyses if more data becomes available.

We conservatively only included health-care utilization for lower respiratory tract infections in the absence of data for other relatively common outcomes caused by RSV, such as upper respiratory tract infections [46]. Similarly, we conservatively did not include quality of life or productivity losses associated with symptomatic RSV infections not requiring a GP visit or hospitalization.

Another factor that makes our estimates potentially conservative is that implementation of vaccination would have no effect on palivizumab use among high-risk infants. Adverse effects of vaccination were not included, because it was assumed that, given the previous troubles while testing a RSV vaccine in children [48], a RSV vaccine will only be registered if it only causes mild local reactions.

Finally, the incidence of RSV hospitalizations was based on the incidence in the Bursa region during a single year. There is some evidence that the RSV incidence follows a biannual pattern in Turkey with seasons with an early strong peak alternating with late seasons with weak activity [49]. Hence, the start of the season and the incidence may differ for other regions of Turkey and different years.

Our findings are similar to studies from high-income countries that concluded that vaccination of infants against RSV has the potential to be cost-effective in the Netherlands [21] and USA [39], or even cost-saving in Spain [50]. It would be interesting to develop and validate a simple generic model to enable further cost-effectiveness evaluations in a range of settings with little extra support, as we are currently doing in the area for influenza. This requires a new project in cooperation with information and communication specialists.

## Conclusion

RSV vaccination of infants and/or pregnant women has the potential to be cost-effective in Turkey. Although using relatively conservative assumptions, all evaluated strategies remained below the threshold of 3 times the GDP per capita. Our study suggests that infant vaccination alone or in combination with vaccination of pregnant women during their third trimester seems to have most potential. When more data become available about vaccine effectiveness against disease and transmission, with more data about the epidemiology of RSV, a dynamic transmission model could be developed to better capture all potential benefits of vaccination.

## Supporting Information

S1 Appendix Schematic representation of the Markov model. (DOCX)
