# Supplementary material for: Potential Cost-Effectiveness of RSV Vaccination of Infants and Pregnant Women in Turkey: An Illustration Based on Bursa Data
Source: PLoS One. 2016 Sep 30;11(9):e0163567. doi: 10.1371/journal.pone.0163567 (PMC5045176; doi:10.1371/journal.pone.0163567)
Supplement: S1 Appendix — (DOCX) [file pone.0163567.s001.docx]

**S1 Appendix. Schematic representation of the Markov**

Figure S1 represents a schematic representation of the Markov model. Note that this is a simplified representation, transition probabilities are calendar month and age-specific. In addition, the transition probability from ‘vaccine immune’ to susceptible is dependent on the vaccination schedule and the age of the infant.


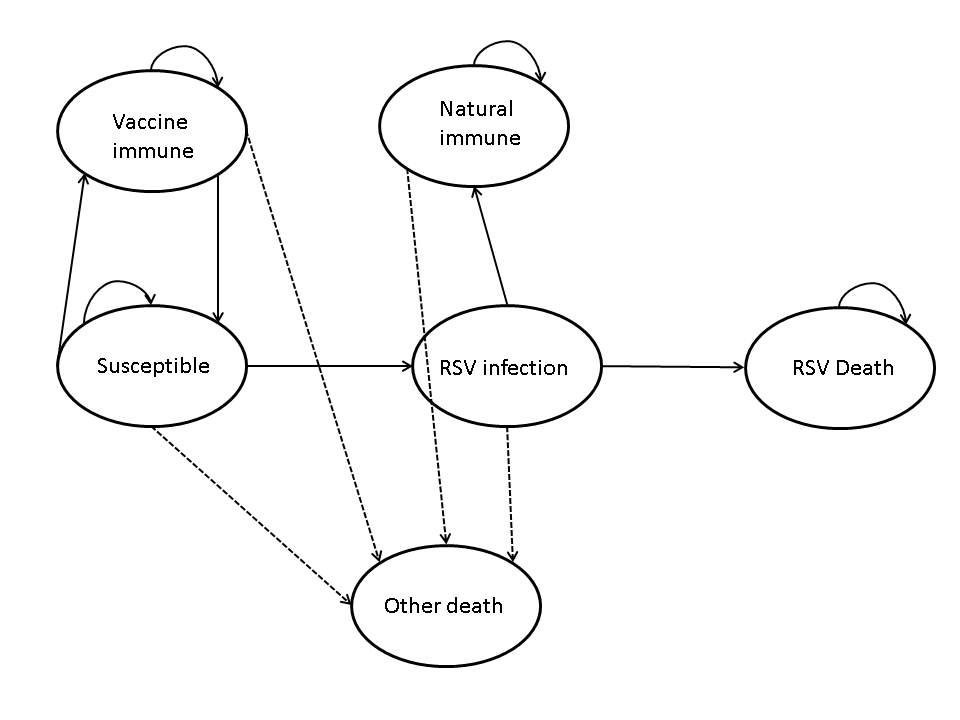


Figure S1. Schematic presentation of the Markov model.

As described in the main text, only RSV infections requiring a GP visits where modelled. Hence, mild (a)symptomatic RSV infections were not included in the model. A proportion of those GP-visits will also lead to a hospitalization, of which a proportion will require an ICU admission and a proportion will die due to the RSV infection. Although re-infection with RSV occurs frequently, re-infections usually have a mild character with symptoms of uncomplicated upper respiratory tract infection [16]. Hence, we assumed that after RSV-related GP visits patients moved to an immune state up until their 2nd birthday. Vaccination reduces RSV-related health care utilization, i.e. RSV GP visits and hospitalizations, and deaths by reducing the amount of children in the susceptible state.
